# How the world’s collective attention is being paid to a pandemic: COVID-19 related n-gram time series for 24 languages on Twitter

**DOI:** 10.1371/journal.pone.0244476

**Published:** 2021-01-06

**Authors:** Thayer Alshaabi, Michael V. Arnold, Joshua R. Minot, Jane Lydia Adams, David Rushing Dewhurst, Andrew J. Reagan, Roby Muhamad, Christopher M. Danforth, Peter Sheridan Dodds

**Affiliations:** 1 Computational Story Lab, Vermont Complex Systems Center, MassMutual Center of Excellence for Complex Systems and Data Science, University of Vermont, Burlington, VT, United States of America; 2 Charles River Analytics, Cambridge, MA, United States of America; 3 MassMutual Data Science, Amherst, MA, United States of America; 4 Faculty of Social and Political Sciences, University of Indonesia, Jakarta, Indonesia; 5 Department of Computer Science, University of Vermont, Burlington, VT, United States of America; 6 Department of Mathematics & Statistics, University of Vermont, Burlington, VT, United States of America; Indiana University, UNITED STATES

## Abstract

In confronting the global spread of the coronavirus disease COVID-19 pandemic we must have coordinated medical, operational, and political responses. In all efforts, data is crucial. Fundamentally, and in the possible absence of a vaccine for 12 to 18 months, we need universal, well-documented testing for both the presence of the disease as well as confirmed recovery through serological tests for antibodies, and we need to track major socioeconomic indices. But we also need auxiliary data of all kinds, including data related to how populations are talking about the unfolding pandemic through news and stories. To in part help on the social media side, we curate a set of 2000 day-scale time series of 1- and 2-grams across 24 languages on Twitter that are most ‘important’ for April 2020 with respect to April 2019. We determine importance through our allotaxonometric instrument, rank-turbulence divergence. We make some basic observations about some of the time series, including a comparison to numbers of confirmed deaths due to COVID-19 over time. We broadly observe across all languages a peak for the language-specific word for ‘virus’ in January 2020 followed by a decline through February and then a surge through March and April. The world’s collective attention dropped away while the virus spread out from China. We host the time series on Gitlab, updating them on a daily basis while relevant. Our main intent is for other researchers to use these time series to enhance whatever analyses that may be of use during the pandemic as well as for retrospective investigations.

## Introduction

Understanding how major disasters affect the wellbeing of populations both in real time and historically is of paramount importance. We especially need real-time measurement to enable policy makers in health systems and government to gauge the immediate situation and evaluate scenarios, and for researchers to model possible future trajectories of social systems. Researchers have demonstrated how characterizing and tracking public discourse of the COVID-19 spread on social media [1–3] can support local authorities’ efforts in response to the global pandemic [4, 5]. Recent studies have also investigated the impact of pre-existing political polarization on discussions related to COVID-19 throughout Twitter’s ecosystem [6], as well as the extent of misinformation on social media [7–9]. Our primary aim here is to generate a particular data stream that may be of help to other researchers: A principled set of *n*-gram time series across major languages used on Twitter and news-relevant for April, 2020. Our work is complementary to extant efforts to enable research on the COVID-19 pandemic [10, 11] by gathering and sharing epidemiological data [12–19], economic data, and internet and social media data [20–24].

In this short piece, we describe how we select languages and *n*-grams relevant to the time period of the present COVID-19 pandemic; show example time series plots for the word ‘virus’ (and its translations), including a visual comparison with COVID-19 confirmed case and death numbers; and describe the data sets, figures, and visualizations for 24 languages that we share online.

## Materials and methods

### Selection of languages and n-grams

We base our curation on our work in two of our previous papers [25, 26], and we draw from a database of approximately 10% of all tweets from 2008/09/09 to present. Our process of obtaining salient *n*-grams for April 2020 comprises two steps. First, we used the language identification and detection tool FastText-LID [27, 28] to evaluate all tweets in our historical archive, finding over 100 languages [25]. Besides analyzing all tweets (AT), we also separately process what we call organic tweets (OT): All Twitter messages which are original. Organic tweets exclude retweets while including all added text for quote tweets. In doing so, we are able to carry through a measure of spreadability for all *n*-grams. The key threshold we use for spreading is the naive one from biological and social contagion models: When an *n*-gram appears in more retweeted than organic material, we view it as being socially amplified. We subsequently extracted day-scale Zipf distributions for 1-, 2-, and 3-grams along with day-scale *n*-gram time series [29]. We preserve case where applicable, do not apply any stemming. We note that the top 10 languages on Twitter comprise 85% of all tweets. Here, we take 24 of the most commonly used languages on Twitter in 2019, with the provision that we are able to parse them into *n*-grams. For the time being, we are unable to reliably parse continuous-based script languages such as Japanese, Thai, and Chinese, the 2nd, 6th, and 13th most common languages. The selected languages comprise two thirds of the daily tweets on the platform. We exclude all tweets not assigned a language with sufficient confidence (an effective 4th ranked collection). In other words, we select the predicted language with the highest confidence score. If the confidence score of our FastText-LID model is less than 25% for a given tweet, then we label that tweet as Undefined (und). We also choose to include Ukranian (29th) over Cebuano (28th) due to a marginal degree of uncertainty for detecting messages written in Cebuano [25]. We list the 24 languages by overall usage frequency in Table 1.

**Table 1:** The 24 languages for which we provide COVID-19 related Twitter time series.

Rank	Language	Code
1	English	en
2	Spanish	es
3	Portuguese	pt
4	Arabic	ar
5	Korean	ko
6	French	fr
7	Indonesian	id
8	Turkish	tr
9	German	de
10	Italian	it
11	Russian	ru
12	Tagalog	tl
13	Hindi	hi
14	Persian	fa
15	Urdu	ur
16	Polish	pl
17	Catalan	ca
18	Dutch	nl
19	Tamil	ta
20	Greek	el
21	Swedish	sv
22	Serbian	sr
23	Finnish	fi
24	Ukrainian	uk

**Fig 1:**
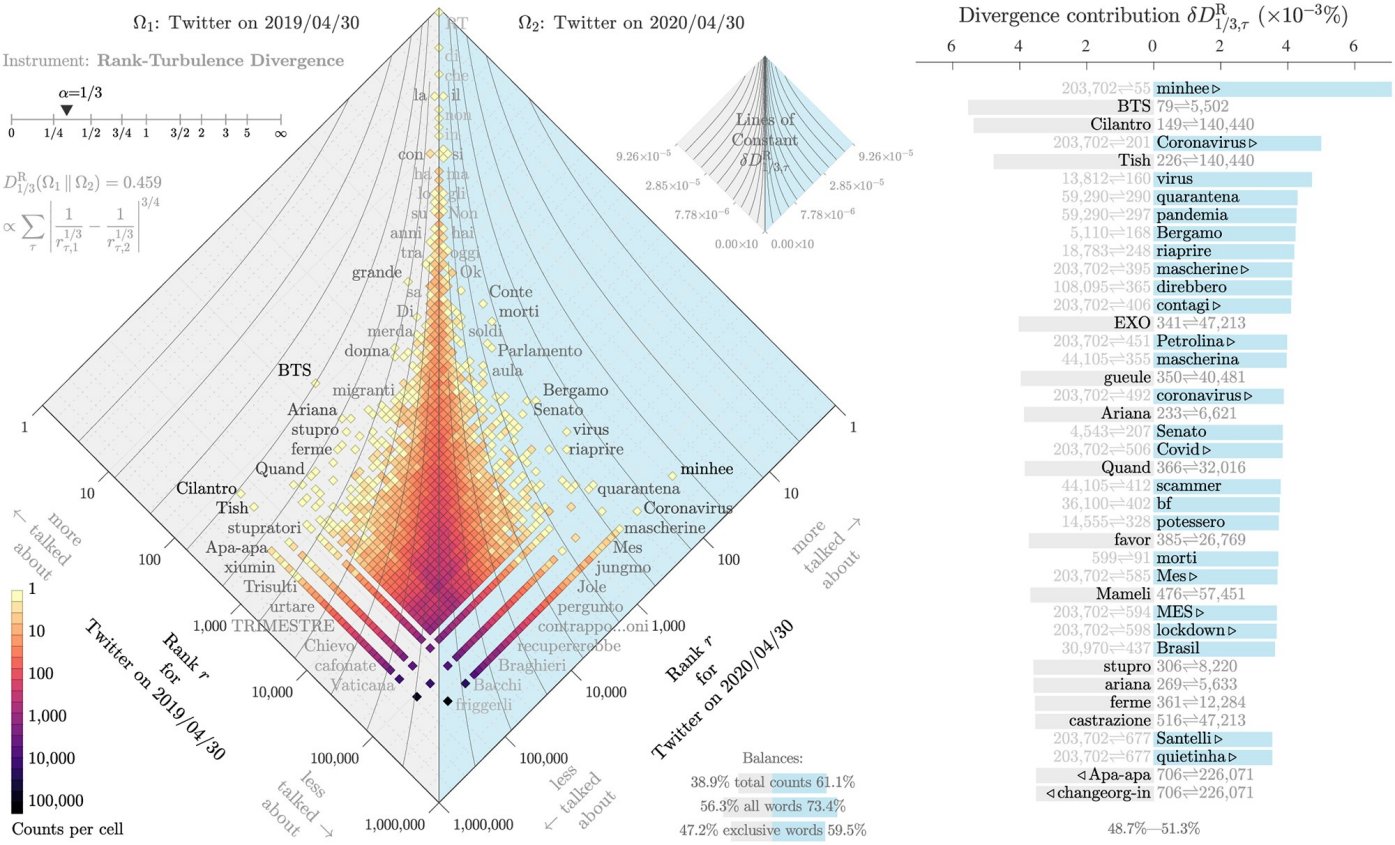
Allotaxonograph using rank-turbulence divergence for Italian word usage on April 30, 2019 versus April 30, 2020. For this visualization, we consider the subset of 1-grams that are formed from latin characters. The right hand sides of the rank-rank histogram and the rank-turbulence contribution list are dominated by COVID-19 related terms. See Dodds *et al.* [26] for a full explanation of our allotaxonometric instrument.

Second, we compare usage of *n*-grams in April of 2020 with April 2019 to determine which *n*-grams have become most elevated in relative usage. We do so by using rank-turbulence divergence [26], an instrument for comparing any pair of heavy-tailed size distributions of categorical data. Other well-considered divergences will produce similar lists. For each language, we take Zipf distributions for each day of April 2020, and compare them with the Zipf distributions of 52 weeks earlier. For an example, we show in Fig 1 an allotaxonograph for Italian comparing 2019/04/30 and 2020/04/30. The main plot displays a rotated 2D-histogram to avoid misinterpretation of causality. We bin *n*-grams logarithmically such that bins located near the center vertical line indicate *n*-grams that are used equivalently on both days, whereas bins on either side highlight *n*-grams that are used more often on the corresponding date. We use rank-turbulence divergence with the parameter *α* set to 1/3 as this provides a reasonable fit to the lexical turbulence we observe [26, 30]. Up to a normalization factor [26], we compute rank-turbulence divergence for each *n*-gram *τ* as follows:

$$\begin{array}{cc} \delta D_{\alpha ,\tau }^{R} & \propto |\frac{1}{r_{\tau ,t_{1}}^{\alpha }}-\frac{1}{r_{\tau ,t_{2}}^{\alpha }}{|}^{1/(\alpha +1)}=|\frac{1}{r_{\tau ,t_{1}}^{1/3}}-\frac{1}{r_{\tau ,t_{2}}^{1/3}}{|}^{3/4}, \end{array}$$

where $r_{\tau ,t_{1}}$ and $r_{\tau ,t_{2}}$ indicate the rank of usage for *τ* at time step *t*_1_ and *t*_2_ respectively. We plot contour lines to demonstrate the scale of rank-turbulence divergence and use divergence contributions of each *n*-gram to compile an ordered set of relevant *n*-grams for each day (see right panel of Fig 1). For ease of plotting, we have further chosen to compare the subset of words containing Latin characters only. Words associated with the pandemic dominate the contributions from 2020/04/30. On the right side of the allotaxonograph, we see ‘Coronavirus’, ‘virus’, ‘quarantina’, ‘pandemia’, ‘Bergamo’, and ‘morti’. We repeat this process for every day in April, and combine divergence contributions for all *n*-grams across these days, and rank *n*-grams in descending order indicating the most narratively dominate *n*-grams for the month of April.

### Data, visualizations, and sites

For each language, and for each of the top *n*-grams we have identified, we extract three day-scale time series starting on 2019/09/01: Daily counts, ranks, and normalized frequencies based on the Eastern Time Zone (ET). Understandably, as the pandemic was unfolding in early 2020, most regional health organizations could not confirm the roots or exact initial date of the first COVID-19 case within their population of charge, with speculations that the virus may have started spreading in late 2019. Therefore, we started our data collection on September of 2019 to cover the last quarter of 2019 and the few months leading to the pandemic spreading worldwide.

The degree to which the pandemic is being discussed on Twitter is of great interest in itself, and our data set will allow for such examination. For the *n*-grams our method surfaces, we observe variations in punctuation and grammatical structures. These variants as well as non-pandemic-related elements may be filtered out for individual languages by hand as may suit interested researchers. We provide a cleaned version of the data set whereby we omit links, handles, hashtags, emojis, and punctuation. We also note that our decision to respect capitalization leads to *n*-grams that some researchers may wish to collapse, and we also provide a case-insensitive version of our data set. We repeat all of the above steps for *n*-grams derived from organic tweets (OT).

We share and maintain all data on Gitlab at: https://gitlab.com/compstorylab/covid19ngrams. We also provide a connected website associated with our paper at: http://compstorylab.org/covid19ngrams/. We show tables of the leading *n*-grams in our data set, as well as example “bar chart races” for the dominant COVID-19 *n*-grams in major languages. Our intention is to automatically update the data set on Gitlab, as soon as we have processed all tweets for a day.

**Fig 2:**
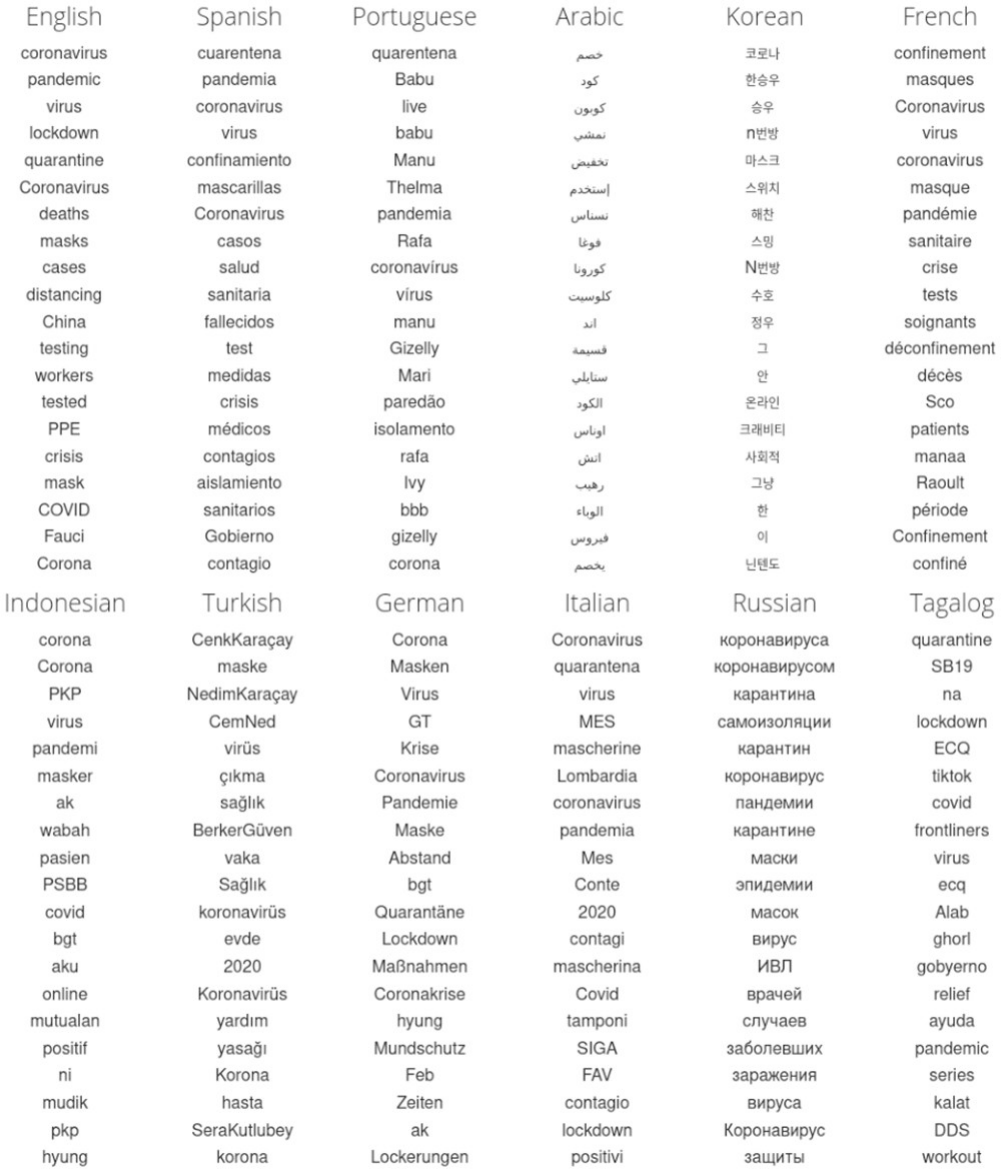
Top 20 (of 1,000) 1-grams for our top 12 languages for the first three weeks of April 2020 relative to a year earlier. Our intent is to capture 1-grams that are topically and culturally important during the COVID-19 pandemic. While overall, we see pandemic-related words dominate the lists across languages, we also find considerable specific variation. Words for virus, quarantine, protective equipment, and testing show different orderings (note that we do not employ stemming). Unrelated 1-grams but important to the time of April 2020 are in evidence; the balance of these are important for our understanding of how much the pandemic is being talked about. To generate these lists we use the allotaxonometric method of rank-turbulence divergence to find the most distinguishing 1-grams (see Sec. Selection of languages and n-grams, Fig 1, and Dodds *et al.* [26]).

**Fig 3:**
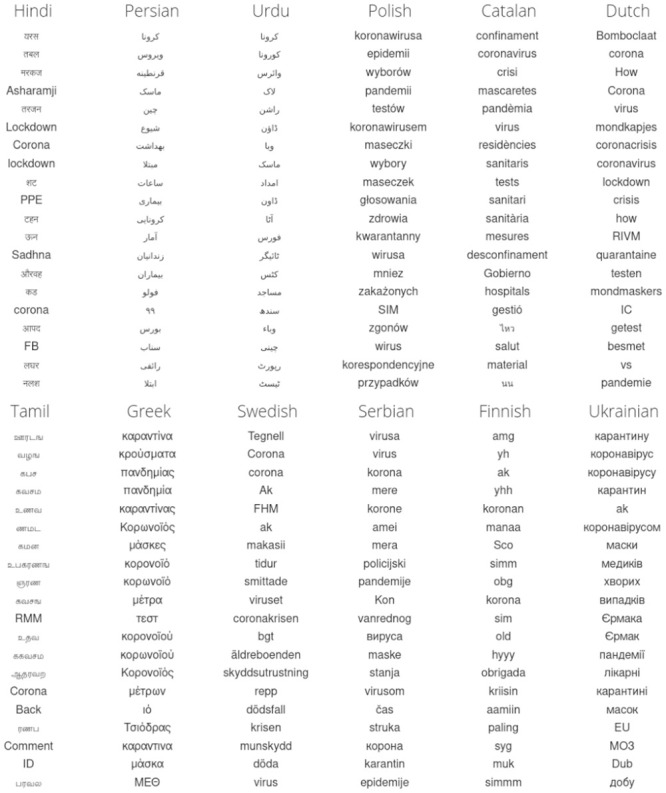
Continuing on from Fig 2: Top 20 1-grams for the second 12 of 24 languages we study for April 2020 relative to April 2019.

We show the resulting top 20 April-2020-specific 1-grams for the 24 languages in Figs 2 and 3. For display, we use the cleaned version, omitting hashtags, handles, emojis, numbers, and punctuation. We also removed all variations of ‘Bomboclaat’ from Dutch. Overall, we see that the lists are dominated by language specific words for coronavirus virus, quarantine, pandemic, testing, and spreading.

In the full, unfiltered data set, some 1-grams such as punctuation represent functional changes in the use of Twitter across languages. The white heart emoji makes the top 20 in a few languages such as English, Arabic, Korean and German. By contrast, and according to the measurements we have used here, the worried face emoji, has become important across many languages in April 2020 relative to April 2019. It would be natural to see this emoji as being pandemic-related but in fact, we see from time series that the worried emoji has slowly being increasing in usage over time for several years (determining the reasons for which we will leave for a separate line of inquiry). All 1-grams are included in the shared raw version of the data sets.

We emphasize that with our approach, we do not explicitly determine whether or not an *n*-gram is relevant to COVID-19. While the pandemic was one of the top stories of 2020 for the majority of countries, there have of course been other major events and moments in popular culture around the world. For example, in March 2020 for the United States, the democratic primary leads to the 1-gram in English Twitter of ‘Biden’ being prominent. Similarly, we see many *n*-grams related to the Big Brother Brazil show in Portuguese, and K-pop in Korean. Further, most languages have a strong degree of geographic specificity (e.g., Finnish for Finland, Portuguese for Brazil), and we have not filtered for precise geo-location. English, Spanish, Arabic, and French are some of the more geographically distributed languages.

## Results and discussion

**Fig 4:**
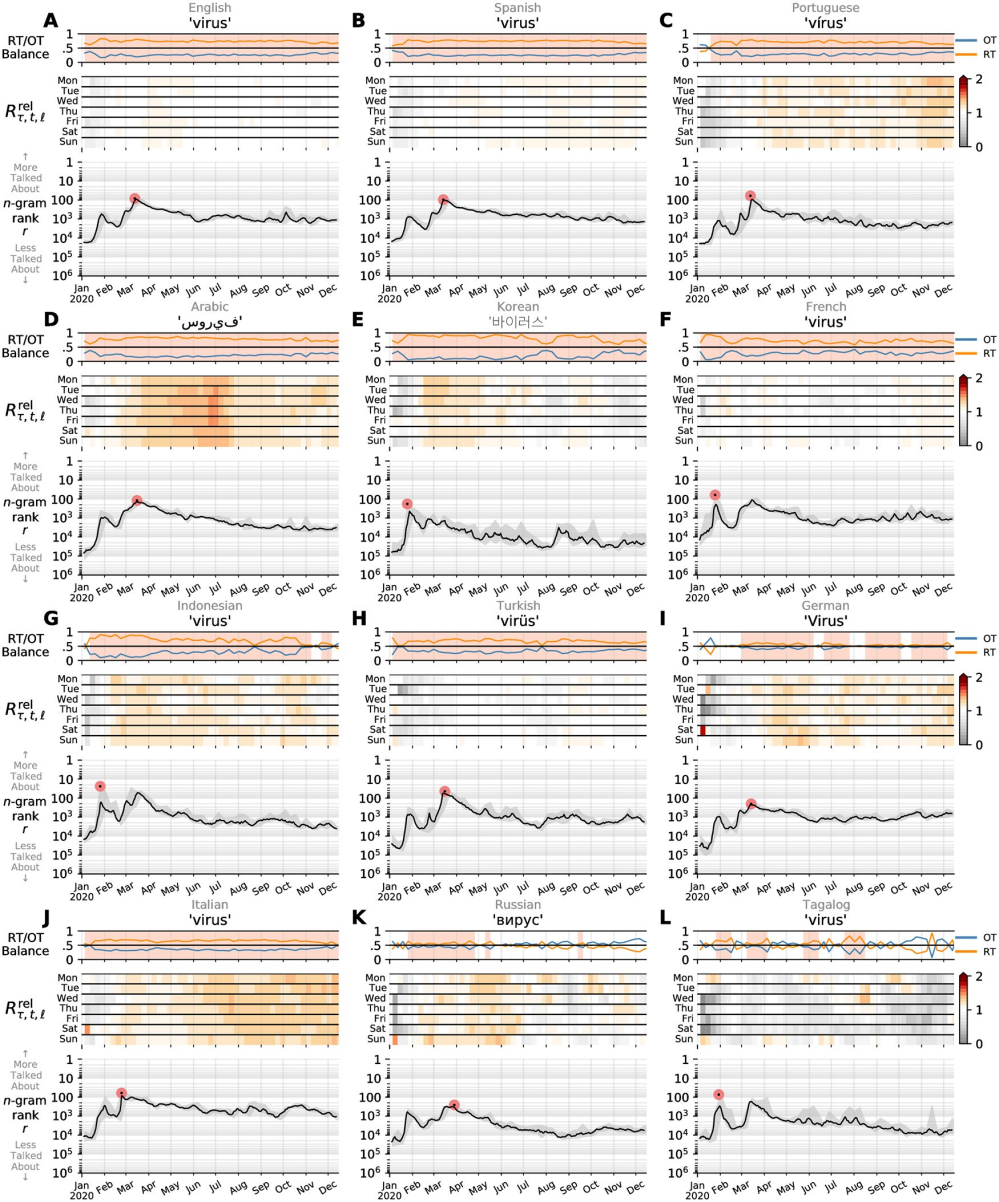
Contagiograms for the word ‘virus’ in the top 12 of the 24 languages we study here. The major observation is that the world’s attention peaked early in late January around the news of an outbreak of a new infectious disease in Wuhan, declining through well into February before waking back up. The main plots in each panel show usage ranks at the day scale (ET). The solid lines indicating smoothing with a one week average (centered). The plots along the top of each panel show the relative fractions of each 1-gram’s daily counts indicating as to whether they appear in retweets (RT, spreading) or organic tweets (OT, new material). The background shading shows when the balance favors spreading—story contagion. See Fig 5 for the next 12 languages, as well as Sec. Results and discussion for general discussion, and Alshaabi *et al.* [29] for technical details of contagiograms.

**Fig 5:**
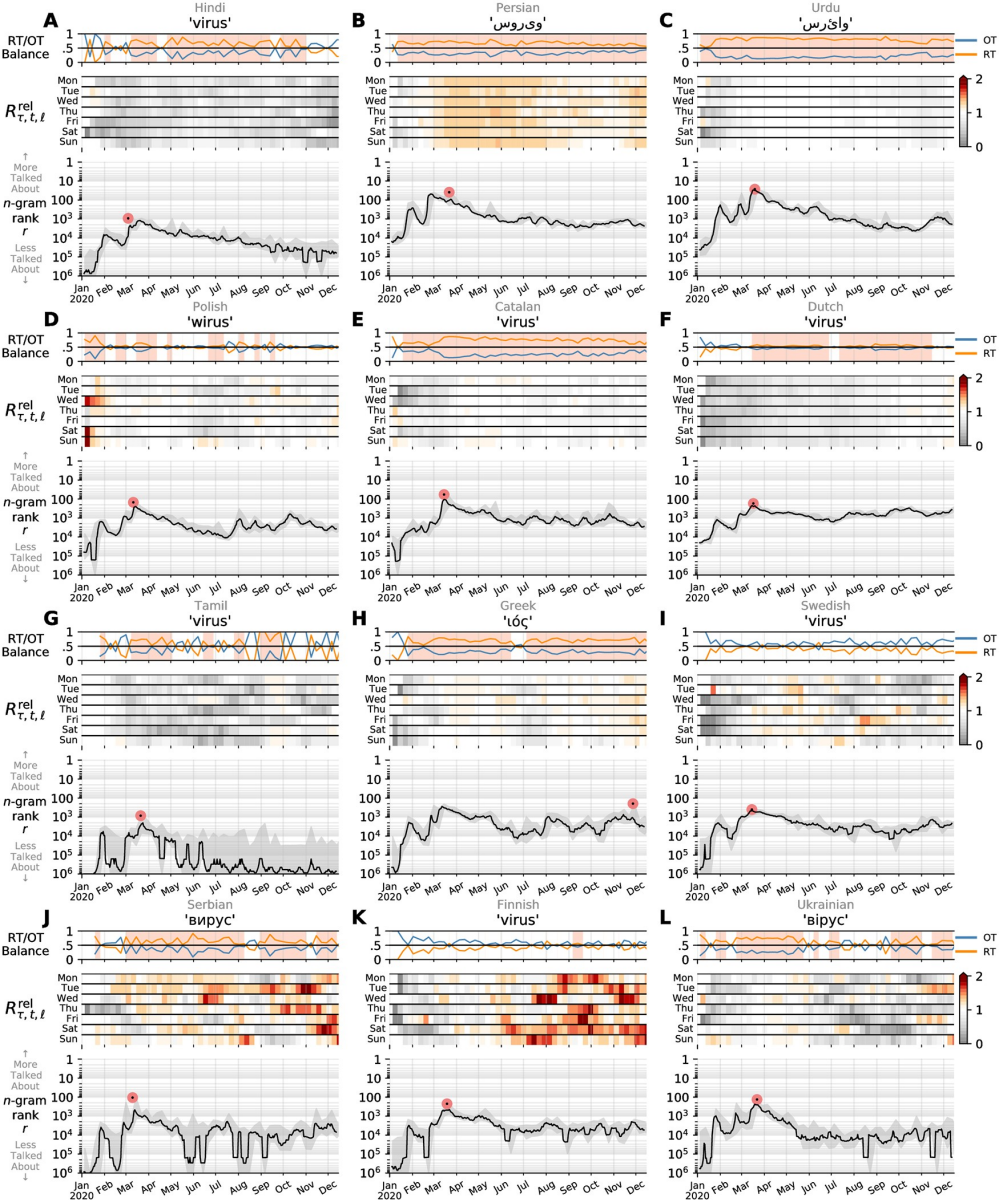
Following on from Fig 4, contagiograms for the word ‘virus’ in the second 12 of the 24 languages. We note that some of these 1-grams are socially amplified over ti me, while others often shared organically.

We briefly consider two sets of sample time series based on our data set. Across Figs 4 and 5, we plot contagiograms [29] for the word ‘virus’ translated as appropriate in to each of the 24 languages. For each language, we display the daily (Zipfian) rank for ‘virus’ in the main panel of each plot. We add a grey background indicating the best and worst rank of each week overlaid by a centered weekly rolling average (black). The pale disk highlights the date of maximum observed rate. In the secondary time series at the top of each panel, we show the relative fraction of 1-gram contained in retweets (RT) versus organic tweets (OT). When the RT/OT balance exceeds 50%, we shade the background to indicate that the 1-gram is being spread (e.g., retweeted) more than organically tweeted. For each contagiogram, we also display a heatmap of the relative amplification of each 1-gram compared to the fraction of 1-grams that are found in RTs on that day. For each day of the week, shades of red indicate higher social amplification, whereas gray shows that the volume of that 1-gram is often shared organically. See Alshaabi *et al.* [29] for technical details of contagiograms, more examples of contagiograms can also be found in S1 and S2 Figs.

Alone, the highest ranks for ‘virus’ show the enormity of the pandemic. While a common enough word in normal times, ‘virus’ has reached into the top 100 ranks across many languages, a region that we have elsewhere referred to as the realm of lexical ultrafame [31]. Normally only the most basic function words of a language will populate the top 100 ranks. In the last few months, we have seen ‘virus’ rise as high as *r* = 24 in Indonesian (2020/01/26), *r* = 27 in Polish (2020/03/11), *r* = 29 in Urdu (2020/03/22), *r* = 44 in German (2020/03/14), and *r* = 83 in English (2020/03/13). In terms of the shapes of the time series for ‘virus’, most languages show a late January peak consistent with the news from China of a novel coronavirus disease spreading in Wuhan. The subsequent drop in usage rate across most of the 24 languages reflects a global decline in attention being paid to the outbreak. The Italian time series for ‘virus’ in Fig 4J shows an abrupt jump about three quarters of the way through February, strikingly just after a drop in RT/OT balance. Persian has a similar shock jump just after midway of February (Fig 4B). We see in Fig 5E that ‘virus’ in Catalan shows no early January peak like most of the other 23 languages, suggesting that even the initial news from China did not have great impact.

One of the major problems we face with the COVID-19 pandemic is the unevenness of testing across the world. South Korea and Iceland have tested early and extensively while the United States’s testing has been uncoordinated and slow to expand. Urdu’s heightened time series for ‘virus’ (Fig 5C) would seem especially concerning given low numbers coming out of Pakistan which, as of 2020/03/24, had reported 1,063 cases and 8 deaths [12]. For Indonesia, where testing has also been limited [12] and with peak attention on Twitter coming in January and early focus on economic issues and evacuation of nationals from Wuhan, a dip in the rank of ‘virus’ in the second half of February is also worrying (Fig 4G).

Countries around the world have adopted different strategies and policies in response to the coronavirus pandemic. While most languages have COVID-19 related terms across the top *n*-grams, some languages also have terms related to other big events happening simultaneously. For example, we see many *n*-grams discussing the democratic primary election in the US. We also find *n*-grams connected to the Big Brother Brazil show in Portuguese, while Korean has many K-pop references. This in part shows that the collective attention of different populations will, indeed, vary depending on the spread of the virus across countries all over the globe for the time period considered in this study. We note, however, that *n*-grams related to the pandemic can still be found in Portuguese showing the initial response to the news about the COVID-19 outbreak as the virus started slowly spreading in Brazil.

**Fig 6:**
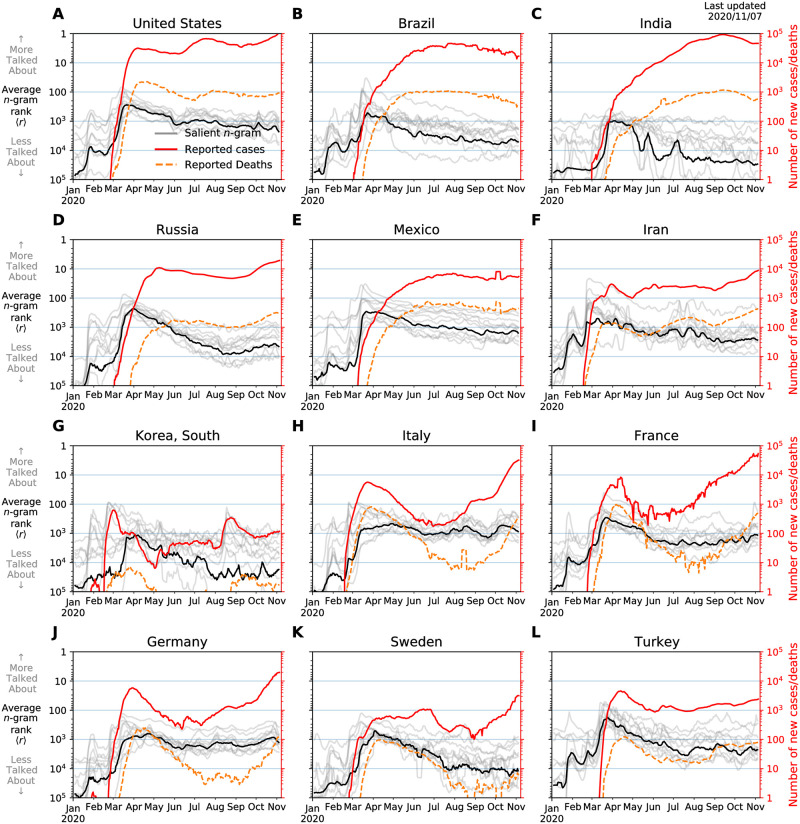
Time series for daily reported case loads and death compared with a list of 10 salient 1-grams for the top language spoken in each country. For each *n*-gram, we display a weekly rolling average of usage ranks at the day scale in gray overlaid by an average of all these 1-grams in black marking their corresponding ranks using the left vertical axis. Similarly, we use the right vertical axis to display a weekly rolling average of daily new cases (red solid-line), and reported new deaths (orange dashed-line). We note that the reported counts are underestimates, more so for cases than deaths, and errors are unknown. We sourced data for confirmed cases and fatalities from Johns Hopkins University Center for Systems Science and Engineering’s COVID-19 project [12]. Starting on 2020/01/22, the project’s data has been collected from national and regional health authorities across the world. The data is augmented by case reports from medical associations and social media posts—these later sources are validated against official records before publication. For the present piece, we use daily summary files for case counts and fatalities, although an API and online dashboard are available for more up-to-date reports.

As one very simple example of comparing our Twitter times series with pandemic-related data, in Fig 6, we present plots of daily reported cases and deaths over time for 12 countries, along with time series for 10 salient 1-grams in the top spoken language for each country. We note that the reported number of cases and deaths are subject to under-reportings. For each country, we use the left vertical axis to plot a weekly rolling average of usage ranks at the day scale for 10 1-grams (gray lines) translated in the top spoken language for each country, while the black solid line shows an average of all these 1-grams. We selected 10 1-grams from the top of each list that are directly related to the coronavirus pandemic to highlight the collective attention around the COVID-19 outbreak. The set of 1-grams we use for each language can be found online at: https://gitlab.com/compstorylab/covid19ngrams/-/blob/master/src/consts.py. Using the right vertical axis, we display a weekly rolling average of daily new cases (red solid-line), and reported new deaths (orange dashed-line).

We see a global surge of attention on Twitter starting mid March through April following the state-wide lockdowns in most countries. Some languages such as Italian and German display a fairly steady level of attention paid to the pandemic. However, the average rank of usage of the selected 1-grams slows down and starts to decay across many languages in April through the summer. In fact, the average rank of usage have dropped an order of magnitude in Indian, Russian, Korean, and Swedish. While the number of new daily cases and deaths are climbing up again, we do not observe the same level of attention reciprocated on Twitter.

## Concluding remarks

We echo our main general observation of how COVID-19 has been discussed through late April 2020: After reacting strongly in late January to the news that a coronavirus-based disease was spreading in China, attention across all but 2 of the 24 languages we survey dropped through February before resurging in late February and through March. We see abrupt shocks in time series as populations shifted rapidly to heightened levels of awareness, particularly in the Italian time series. In the time series for ‘virus’, we see two and sometimes three peaks of attention in the space of just a few months. Our hope is that our collection of Twitter *n*-gram time series that are especially relevant to April 2020 will be of benefit to other researchers. The time series we share will, in part, reflect many other aspects beyond mentions of ‘virus’, which we have only briefly explored here. Possible topics to investigate include washing (including the soap and microbe emojis), testing, serology, vaccine, masks and protection equipment, social and physical distancing, terms of community support versus loneliness and isolation, closures of schools and universities, economic problems, job loss, and food concerns.

We repeat that the lists we provide are meant to represent the important *n*-grams of April 2020, and we urge a degree of caution in the use of the data set. As we have indicated above, our lists of *n*-grams contain some peculiarities that will not be directly relevant to COVID-19. Entertainment (e.g., movies, celebrities, and K-pop) and sports (football along with sports in the United States) are standard fare on Twitter when no major events are taking place in the world. The extent to which these aspects of Twitter are submerged as pandemic related *n*-grams rise is of interest.

Finally, while we have been able to identify languages well, geolocation is coarse and at best will be at the level of countries. The strength of geolocation for our time series will depend on the degree of localization of a given language as well as Twitter user demographics. We leave producing *n*-grams with serviceable physical location as a separate project.

## Supporting information

S1 Fig Examples of 1-gram time series. A collection of salient 1-grams across the top 12 languages for April 2020 relative to April 2019.
(TIF)

S2 Fig Examples of 2-gram time series. A collection of salient English 2-grams for April 2020 relative to April 2019. We note a rich and wide range of cultural, geopolitical and socioeconomic references in the selected 2-grams.
(TIF)
