# Supplementary figures and images for: How the world’s collective attention is being paid to a pandemic: COVID-19 related n-gram time series for 24 languages on Twitter
Source: PLoS One. 2021 Jan 6;16(1):e0244476. doi: 10.1371/journal.pone.0244476 (PMC7787459; doi:10.1371/journal.pone.0244476)

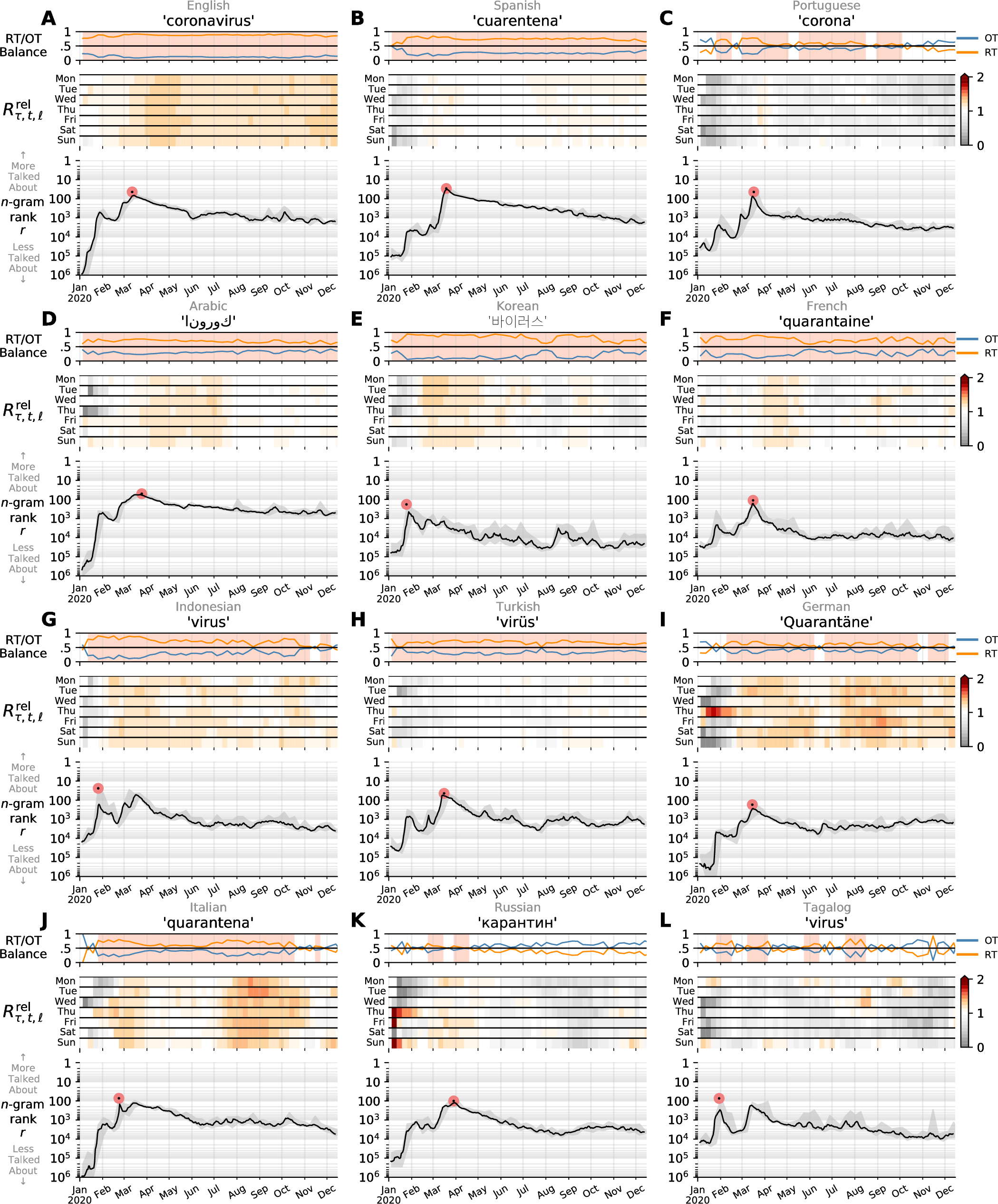

Supplement: S1 Fig — A collection of salient 1-grams across the top 12 languages for April 2020 relative to April 2019. (TIF) [file pone.0244476.s001.tif]

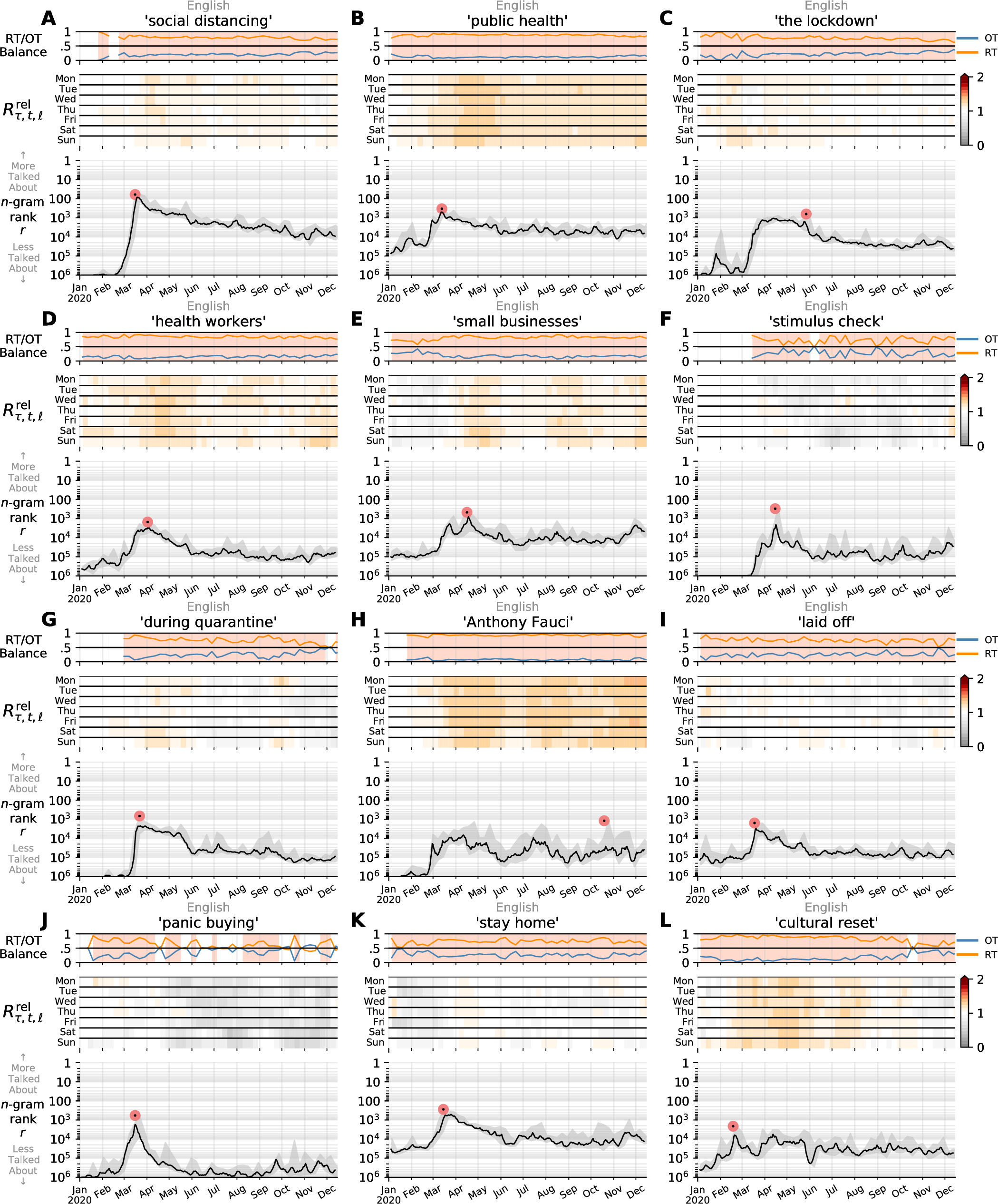

Supplement: S2 Fig — A collection of salient English 2-grams for April 2020 relative to April 2019. We note a rich and wide range of cultural, geopolitical and socioeconomic references in the selected 2-grams. (TIF) [file pone.0244476.s002.tif]
